# Coercive Control and Mother–Child Relationships: Exploring Mothers’ Experiences of Talking With Their Children About the Abuse

**DOI:** 10.1177/08862605251329498

**Published:** 2025-03-31

**Authors:** Sarah Kelly, Olga Luzón

**Affiliations:** 1 Royal Holloway University of London, Egham, UK

**Keywords:** coercive control, domestic abuse, mother–child relationships, mother–child communication, interpretative phenomenological analysis

## Abstract

Coercive control (CC) is a severe and relentless form of domestic abuse whereby perpetrators often directly target and undermine women’s relationships with their children. A “conspiracy of silence” can surround the abuse, with mothers and children not speaking to each other about what has happened, which has been described as negatively impacting the mother–child relationship. Our understanding of how mothers experience conversations with their children about their shared experiences of CC is limited. The current study, therefore, aimed to explore: (a) mothers’ experiences of their relationships with their children in the context of CC, and (b) mothers’ experiences of talking with their children about the CC. Eleven interviews were conducted between July and November 2023 with mothers who had experienced CC who had been separated from the perpetrator/father for at least 1 year. Data were analyzed using interpretative phenomenological analysis. Five group experiential statements were constructed, with 12 group-level subthemes. Two group experiential statements pertained to women’s experiences of the mother–child relationship, one to how women experienced conversations about the abuse as positive for the mother–child relationship, and two to the facilitators and barriers that women experienced when navigating these conversations. The findings are discussed in relation to four key implications: the importance of joint mother–child interventions; the need for education and training for professionals; education in schools; and urgent reform to UK family courts.

## Introduction

Coercive control (CC) is a severe and relentless form of domestic abuse that is primarily perpetrated by men against women and children (Myhill, 2015). Evan Stark coined the term in his 2007 book to describe a pattern of oppressive behaviors whereby the perpetrator controls, intimidates, degrades, and isolates their victim with the intention of gaining complete control over their life. While CC may involve physical violence, Stark (2007) argues that focusing on isolated incidents of violence is inappropriate and unhelpful as violence in these situations, if present at all, tends to be repetitive and low-level; its significance lies in obtaining control rather than its injurious effects. Similarly, Katz (2016, 2022) advocates for a move away from a physical incident model of domestic abuse, toward recognizing patterns of coercive and controlling behaviors, which may or may not involve physical violence.

The UK government criminalized CC within the Serious Crime Act (2015), which has since been amended in April 2023 stating that controlling or coercive behaviors that take place *after* a relationship has ended will also now be recognized as a criminal offence. This is in recognition of the sustained or increased CC that women often experience post-separation (Spearman et al., 2023; Tutty et al., 2023), with the period immediately after leaving representing the highest risk of homicide (Adhia et al., 2019; Campbell et al., 2003). The post-separation context is particularly complex for women who have had children with the perpetrator, where the family court and legal systems can not only fail to identify CC but can in fact facilitate ongoing abuse and control over mothers and children through court-ordered contact with the perpetrator/father (Douglas, 2018). Another dangerous aspect to family court proceedings concerns the threat of being accused of “parental alienation” (PA), a term that has been used to discredit and silence mothers’ allegations of domestic abuse by accusing her of attempting to “alienate” the children from the father (Birchall & Choudhry, 2022; Meier, 2020). PA has been discredited within the scientific community, described by the United Nations as an “unfounded and unscientific concept” (United Nations, 2023). The European Parliament has also called on member states to discourage or even prohibit the use of PA in family court proceedings due to its use by abusive fathers as a continuation of power and control (European Parliament, 2022). Despite this, PA continues to be a powerful weapon within family courts, with detrimental consequences for custody arrangements (Birchall & Choudhry, 2022; Meier, 2020).

Previous research highlights a number of ways in which perpetrators of domestic abuse deliberately attempt to undermine the mother–child relationship, such as by emotionally abusing women in front of children, thereby belittling them in the eyes of their children or even encouraging children to join in (Mullender et al., 2002). Furthermore, the environment of sustained hostility created by perpetrators restricts the space that women and children have to build their relationship (Buchanan, 2018). Domestic abuse results in unique challenges to parenting, with mothers describing the increased sense of responsibility placed on them to look after their child while simultaneously experiencing a loss of control over their parenting due to the abuse and control by their partner (Lapierre, 2009, 2010). Despite these challenges, not all mother–child relationships are invariably and irreversibly damaged by domestic abuse, with previous research highlighting that, while some mother–child relationships are harmed by the abusive behavior of the perpetrator, others remain close and supportive (Buchanan, 2018; Humphreys et al., 2006). Previous research has explored the many ways in which women protect and support their children in situations of domestic abuse, such as by creating stability and consistency for children, ensuring quality time together to do fun activities, listening to children, and having conversations about healthy relationships (Fogarty et al., 2019; Lapierre, 2009, 2010).

Early qualitative research with women and children survivors demonstrated that a “conspiracy of silence” often surrounded their experiences of the abuse, with women describing their lives during the abusive relationship as “dominated by secrecy, silence and fear” (Mullender et al., 2002, p.175). Women had often not spoken with their children about the abuse in an attempt to protect them from the reality of what had been happening, due to explicit threats of violence if they did so, or concerns over how to discuss the abuse with children in an age-appropriate way (McGee, 2000; Mullender et al., 2002). Children, in turn, did not then initiate these conversations, despite often being far more aware of the abuse than their mothers realized and desperately wanting to be able to talk about and understand what had been happening (McGee, 2000; Mullender et al., 2002). This “conspiracy of silence” sometimes persisted post-separation, continuing to harm the mother–child relationship as children remained feeling unclear about what had happened (Mullender et al., 2002). McGee (2000) describes how “communication appeared to be the key in differentiating those young people who blamed their mother and those who felt that their shared experiences of violence had brought them closer” (p. 83).

Several studies have since furthered the understanding of the barriers and facilitators of open mother–child communication about domestic abuse (Humphreys et al., 2006, 2011; Insetta et al., 2015; Moulding et al., 2015). Humphreys et al. (2006, 2011) developed activity packs aimed at strengthening mother–child relationships post-separation by providing opportunities to talk about feelings and experiences from the past. However, the authors noted several barriers to supporting mother–child communication, such as organizations providing separate mother and child domestic abuse services. Furthermore, the refuge setting was often not the most stable atmosphere for supporting conversations between women and children about the abuse, with mothers and children arriving at refuges at a time of crisis when they were not yet safe from the abuser. At a wider level, Moulding et al. (2015) conceptualize women’s silence with their children as a form of maternal protectiveness, understood in the context of gendered mothering discourses where responsibility lies with the mother for keeping the family together, putting her own needs aside for the good of the family unit. There is limited consideration in previous research of the facilitators of these conversations, which may in part be due to many women in previous research having never had these conversations with their children (Insetta et al., 2015; Moulding et al., 2015).

Previous studies are limited in a number of ways. First, samples are limited by a reliance on recruiting via domestic abuse refuges (Humphreys et al., 2006, 2011; Insetta et al., 2015), which only allows a limited focus on mother–child communication across the post-separation period as families are likely to be accessing refuges at the point of separation. Furthermore, the experiences of physical abuse and need for safety may be higher than for other women and children in the community. Second, previous research in this area tends to focus on mother–child communication about incidents of physical violence, which is problematic in several ways. Talking with children about a discrete violent act is a different experience to talking with children about patterns of coercive and controlling behaviors, which may or may not involve physical violence. Furthermore, as CC tends to continue and even intensify post-separation (Spearman et al., 2023; Tutty et al., 2023), for many women and children, talking about incidents of physical violence at home may not be applicable at all to their child’s experience of CC, which may have primarily involved post-separation abuse from perpetrators/fathers.

Overcoming the aforementioned limitations, Katz (2022) explored the impact of CC on mothers and children, and on the mother–child relationship, by interviewing 15 mothers and 15 children between 2011 and 2012. While the research is focused more generally on how mothers and children are affected by CC, and their agentic strategies of resistance, Katz (2022) draws attention to the importance of mother–child communication about the abuse when discussing mothers’ and children’s recovery strategies. Similar to previous research, mothers and children reported that they often did not talk about the CC while living with the perpetrator/father, with mothers reporting that their silence was part of an emotional survival strategy, and children reporting that they did not share their feelings with their mothers as they did not want to upset them (Katz, 2022). Post-separation, the mothers and children in this study reported that being able to talk openly about the CC was vital for improving the mother–child relationship. Women and children described how talking about the abuse helped to shift feelings of blame and anger away from themselves and each other and toward the perpetrator/father (Katz, 2022). She discusses how perpetrators of CC use tactics of denial, minimization, justification, and gaslighting to deliberately distort reality, creating a version of events where they “did not really do anything wrong” or where they were not really responsible for their behavior, which makes mother–child communication about the abuse both so difficult and yet so important in order to break free of this distorted reality.

Katz’s (2022) research highlights several important areas for further study. First and foremost, while Katz (2022) emphasizes mother–child communication about the abuse as an important part of their recovery, there remains a gap in our understanding of how mothers *experience* these conversations with their children, which is important for practitioners supporting women and children survivors. Second, approximately half of Katz’s (2022) participants had accessed specialist mother–child domestic abuse recovery programs, and many commented on how helpful these were in informing children about domestic abuse, which facilitated mother–child communication about the past. Katz (2022) notes how the use of these programs is much less than 50% in the general population of survivors. Therefore, it is likely that, for many women and children without access to these programs, their experiences of talking about the CC may be quite different.

### Aims of the Current Study

Given the proposed importance of mother–child communication about domestic abuse in terms of strengthening mother–child relationships post-separation (Katz, 2015, 2022; McGee, 2000; Mullender et al., 2002), and the lack of research focused on conversations about CC specifically, the current study aimed to explore: (a) mothers’ experiences of their relationships with their children in the context of CC, and (b) mothers’ experiences of talking with their children about the CC.

## Method

### Design

This study used a qualitative design employing the use of semi-structured interviews and data were analyzed using interpretative phenomenological analysis (IPA) based on Smith et al. (2022). IPA is said to involve a “double hermeneutic” as the researcher is trying to make sense of the participant trying to make sense of their personal and social world.

### Participants

Participants were mothers who had experienced CC alongside their child/children from either their child/children’s father or an ex-partner; who had been separated from the perpetrator/father for at least 1 year; and whose child/children were minimum age 4 at the time of the interview. Participants were recruited via social media and third-sector domestic abuse organizations. Efforts were made to recruit people of the global majority through contacting 42 organizations specifically supporting women from minoritized backgrounds. However, all participants were eventually recruited via social media. The final sample was comprised of 11 participants; demographic characteristics are presented in Table 1.

**Table 1.** Characteristics of Sample.

Pseudonym	Age	Ethnicity	Age of Child/Children	Length of Time Since Separation (years)	Duration of Relationship (years)	Relationship Between Children and Perpetrator	Custody & contact arrangements
Elizabeth	48	White British	10 (female) 13 (male)	1	31	Biological father	No current contact, undergoing family court proceedings to determine contact arrangements
Gemma	44	White British	5 (male) 7 (male)	6	4	Biological father	No current contact, letterbox contact four times a year
Dilys	44	White British	6 (male) 17 (female) 19 (male) 23 (female)	16	6	Biological father to 17- and 19-year-old/stepfather to 23-year-old	No current contact, no contact order past three years
Nicola	51	White British	10 (female) 26 (male)	9	5	Biological father to 10-year-old/stepfather to 26-year-old	Regular court-ordered contact
Elena	55	White Spanish	16 (male) 19 (female)	11	13	Biological father	No current contact, no contact order since 2017 ordering only telephone or supervised visits
Imogen	42	White British	15 (female) 17 (male)	12	8	Biological father	Regular contact
Kara	44	White British (Welsh)	5 (female) 11 (male) 24 (male) 27 (female)	20 (first relationship), 11 (second relationship)	10 (first relationship), 2 (second relationship)	Biological father to adult children; biological father to 11-year-old/stepfather to adult children	First relationship – no current contact, father lives abroad Second relationship – regular court-ordered contact
Charlotte	51	White British	11 (male)	4	11	Biological father	Regular court-ordered contact
Mia	39	White North American	10 (female)	7	4	Biological father	Regular court-ordered contact
Tamara	31	White Ukrainian	5 (female)	5	3.5	Biological father	Regular court-ordered contact
Annie	57	White British	18 (female) 20 (male) 22 (male)	9	15	Biological father	All adult now, post-separation regular contact with father via social services contact schedule

### Data Collection

Eleven interviews were completed between July and November 2023; two took place face-to-face and nine over video call. Each interview lasted approximately 90 min. Experts by experience were consulted regarding the design of the recruitment materials and interview schedule. The interview focused on participants’ experiences of their relationships with their children (e.g., “How would you describe the relationship?”), as well their experiences of talking with their children about the CC (e.g., “Can you give any examples of what these conversations looked like?”). The interview ended with consideration about the future and positive aspects of the women’s experiences (e.g., “What are your hopes for the future in terms of your relationship with your children? Overall, what do you think has most helped your relationship with your children?”).

Participants provided written consent for research participation, which included consent to contact the participant’s GP should issues around safety arise. Throughout the study, it was emphasized that participation was entirely voluntary, and participants were free to withdraw at any point. After the interview, participants were offered a debrief and sent a list of contact details of domestic abuse and mental health support services. Participants received a follow-up call from the researcher within a week of participation.

### Analysis

The interviews were audio-recorded and transcribed. Following initial reading and re-reading, exploratory noting was used to identify specific ways in which participants talked about the key experiences of interest. Experiential statements were then constructed, and clusters of experiential statements were outlined and given a name to represent personal experiential themes (PETs). An overall table of PETs, subthemes, and illustrative quotes was constructed for each participant. Once each transcript had been analyzed, all of the PETs were examined to create a set of group experiential themes (GETs), with group-level subthemes.

## Results

**Figure 1.**
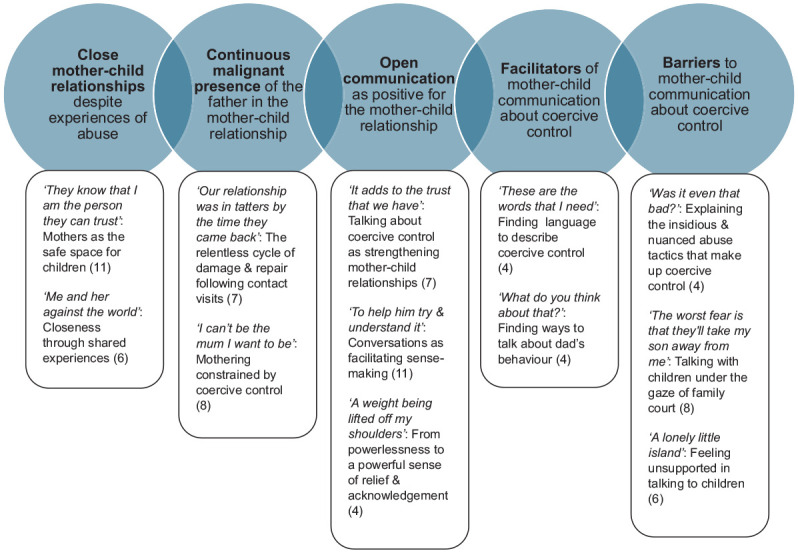
Group experiential statements and group-level subthemes.

The analysis produced five group experiential statements (GETs), with 12 group-level subthemes (Figure 1). Numbers in brackets show the number of participants contributing to each subtheme.

### GET 1: Close Mother–Child Relationships Despite Experiences of Abuse

Participants experienced close relationships with their children, which is discussed in relation to trust and a sense of closeness through shared experiences.

#### Subtheme 1: “They Know That I Am the Person They Can Trust”: Mothers as the Safe Space for Children

There was a shared experience among participants in relation to being the safe and secure parent figure amid the instability resulting from the father/perpetrator’s CC, which mothers experienced as conferring a sense of trust.

*“They know they're important. And they know they're my number one priority. So I think they feel very secure with me. I don't, they might not feel secure about everything else that's around us, but I think they trust me and know that they're loved.”* (Gemma)

Gemma contrasts the sense of security she perceives her children to experience with her against the backdrop of insecurity, which characterizes the rest of their lives. This perceived sense of security and trust seems to be linked to Gemma’s efforts to prioritize her children above all else, an experience echoed in Elena’s account.

*“I think they know that I am the person they can trust because I have always been on their side. . .I am the, well, the safe net for them.”* (Elena)

Elena’s description of being the *“the safe net”* is similar to Kara’s description of being *“the safe space”* for her son, as well as Tamara’s description of being *“the secure parent”* for her daughter. The use of “the” across all these quotes is interesting; “the” is a definite article used when there is only one of something, possibly emphasizing how mothers are the *only* safe and secure attachment figure. There seems to be a shared experience of mother–child closeness as inextricably linked to providing a sense of safety and security for children, not to be underestimated when parenting in the context of CC.

#### Subtheme 2: “Me and Her Against the World”: Closeness Through Shared Experiences

Several participants reflected on feeling as though their shared experiences of CC had brought them closer with their children.

*“I think in that way we are [closer], whether we purposefully are closer because of that, but I think we just automatically are because we're going through the same thing.”* (Charlotte)

*“It was quite a lonely time for us. But it was like me and her against the world. It was, it really was, it was so, so sweet. And I really miss it so much.”* (Mia)

However, it is important to note that this closeness was not always experienced as wholly positive. Nicola reflects on the authenticity that exists between her and her son, experiencing this as both positive and negative for their relationship due to the vulnerable position he had to see her in. This aligns with Mia and Tamara’s experiences too; while they felt a strong closeness with their daughters, this sense of being *“me and her against the world”* (Mia) perhaps comes at the cost of feeling disconnected from wider support.

*“In some ways it’s brought us closer because I think there has had to be an honesty about difficult things that, um, we wouldn’t have had to do. I think in other ways. . .I think sort of him seeing me so vulnerable and so unable to make it better, make it work, makes me feel like I've failed him, um, and yeah, but again, it sort of comes with sort of there’s an authenticity. . .so in some ways I think that’s a good thing.”* (Nicola)

### GET 2: Continuous Malignant Presence of the Father in the Mother–Child Relationship

It was a universal experience among the women in this study that the CC continued post-separation, for several women over 10 years, with most reporting that the abuse intensified after separating. The continuous malignant presence of the father in the mother–child relationship is discussed in relation to the relentless cycle of damage and repair following contact visits and the ways in which the CC constrained women in their parenting.

#### Subtheme 1: “Our Relationship Was in Tatters by the Time They Came Back”: The Relentless Cycle of Damage and Repair Following Contact Visits

Many mothers reflected on the experience of having to send their children for court-ordered contact with the abusive father, and the resulting relentless cycle of having to re-build the mother–child relationship following the damage inflicted by the father.

*“We had such a tight little unit, but it would be, you know, maybe three hours from him, and it was chaos. Our relationship was in tatters by the time that they came back. And I had to then build that all up again.”* (Kara)

*“When they came back, they would be very hostile towards me and instead of running into my arms to greet me, they would be almost wary of me. . .it was almost like they didn't trust me. . .And then I would have a few days with them, and things would return to normal. And then if they would see him again. . .it would all start again.”* (Imogen)

Many mothers reflected on feelings of helplessness as contact with the father had been court-ordered, rendering them powerless to stop it. However, many mothers also showed agentic strategies whereby they were able to repair the mother–child relationship. Kara described *“serious damage control”* following contact visits, similarly to Tamara highlighting her tireless efforts to rebuild and repair her relationship with her daughter:

*“I try to get space with Anna and reassure her. . .She just shuts down to me, she doesn't talk to me. . .But I just sit there and I just tell her, you know, 500 times, I tell her that I love her and that that's not true, that I'm sorry daddy feels that way.”* (Tamara)

#### Subtheme 2: “I Can’t Be the Mum I Want to be”: Mothering Constrained by CC

Several women reflected on their identity as a mother and how the abuse constrained their ability to parent in the way they wanted to.

*“You want to be there for your child, you want to listen to your child, you want to show your child that they have someone to confide in, that they are believed, that they can be heard, and you are that person. But then at the same time, you're kind of stuck in this place that if you do that, you are criticised. . .I just feel that I'm torn all the time between what I really want to be doing with my son in the way I behave, and the way I have to behave because there's a court order in place. . .I can't be the mum I want to be, I can't do anything because of the family court hearing.”* (Charlotte)

Charlotte reflects on the values she holds central to parenting, before describing how the father’s ongoing control via the family courts keeps her from channeling these values. Several other women experienced this constraint in relation to not being able to be fully present with their children as a result of ongoing post-separation abuse.

*“It's all affecting my relationship with her because I'm not 100% present.”* (Tamara)

Tamara reflects on the anxiety she feels, which prevents her enjoying quality time with her daughter, echoed in Charlotte’s description of parenting *“consistently on high alert”* and Mia’s experience of feeling *“hypervigilant”* in her parenting post-separation. Gemma powerfully captures how the CC fundamentally changed her entire experience of her relationships with her sons and her identity as a mother and as a person:

*“I really feel like he robbed me of my experience of motherhood, and I feel like he's robbed the boys of having me. And I know they don't know anything but what I am now, but I know that that's not, I don't want to say it's not who I am, because I guess it is who I am, but it's not who I feel like I should be or who I want to be. So our entire relationship has been different to how I would have hoped and wanted it to be in so many ways.”* (Gemma)

### GET 3: Open Communication as Positive for the Mother–Child Relationship

Overall, participants spoke positively about their experiences of talking with their children about the CC, although the balance of knowing how much to share in an age-appropriate way was a dilemma shared by all. Mothers experienced these conversations as generally positive for the mother–child relationship in terms of enhancing trust, facilitating sense-making, and facilitating a powerful sense of relief and acknowledgement.

#### Subtheme 1: “It Adds to the Trust That We Have”: Talking About CC as Strengthening Mother–Child Relationships

A number of mothers perceived talking about the abuse as enhancing trust between them and their children.

*“I think [talking about the CC] made us closer. I think we would be close anyway, but I think it just like, they can see that I'm being honest with them. So I think they, it adds to the trust that we have because they know that I'm telling them things.”* (Gemma)

Many mothers spoke about valuing honesty within their relationships with their children and feeling as though openness and honesty were important indications of closeness. Therefore, talking honestly about the abuse may represent an important way in which mothers move toward regaining control over parenting in the way that they want to and more in line with their parenting values. In answering what has most helped the mother–child relationship, some mothers experienced talking about the abuse as being the most helpful act in strengthening their relationships with their children post-separation.

*“Definitely being able to talk to them about it and being able to be open and honest has had the most impact”.* (Imogen)

#### Subtheme 2: “To Help Him Try and Understand It”: Conversations as Facilitating Sense-Making

All the mothers in this study reflected on the conversations they had had with their children about CC as facilitating a shared understanding of the abuse. There was an acknowledgement that children are very much aware of the abuse and they need a way to make sense of these experiences.

*“More to help him try and understand it. . .Show me any kid his age [11], and they are aware of everything. You don't have to be showing them stuff. Of course they're aware of it. They're aware.”* (Charlotte)

*“She's described it to me as like, ‘it's like a magic trick what they what they do, mum. It's like they know how to do magic on your brain’. This is something that she came up came out with, like back in year three, year four. And that's sort of when I started to try, I tried to explain to her what the real words were.”* (Mia)

Several women also emphazised to children that the abuse was not their fault, and as such these conversations represent a protective act from mothers as they attempt to facilitate sense-making for children in a way which protects against possible feelings of guilt.

*“I think it is an explanation that it was not their fault, that this was trying to get control and trying to cause harm to me.”* (Elena)

It is also important to highlight the perceived harms in talking to children about CC. Several mothers, while agreeing that talking to children in some way can be helpful, felt that these conversations had the potential to do more harm than good if too many details were shared, such as Annie’s reflection that it would *“add to their distress”* if her children knew the whole truth about their father. Elena also expressed reluctance to discuss details of the abuse from when children were very young that they may not remember.

#### Subtheme 3: “A Weight Being Lifted Off My Shoulders”: From Powerlessness to a Powerful Sense of Relief and Acknowledgement

There was a shared experience across several mothers in this study of a sense of relief in having spoken with children about the abuse.

*“I asked them, ‘what do you think about how he treats me?’. And then one of them, I don't know who, said, ‘bad’. And the other one said, ‘very bad’. So they knew. . .It's the type of thing that you are thinking how to do it and it was so simple that it was a big relief that they accepted that and they understood that.”* (Elena)

*“I think for me a relief and I sort of felt a flood of happiness that I was, he was finally able to, to acknowledge these things after years of being wary of speaking about it. . .It's almost like it’s set me free in a way, and that probably sounds cliche and cheesy, but that's almost how it felt. It was almost like, you know, after 12 years of a weight being lifted off my shoulders that ah, finally, we can talk about it.”* (Imogen)

Imogen’s experience of talking with her teenage son is particularly powerful following 12 years of post-separation abuse where she was continuously spoken about in a derogatory way to him by the father. This idea of conversations as empowering is also reflected in Elizabeth’s account, where the act of talking openly with her children is in itself experienced as a powerful act of defiance against the father’s control.

*“I think it's been positive [talking about the CC]. I think that they can see that I can now speak freely in a way that just wasn't possible before.”* (Elizabeth)

### GET 4: Facilitators of Mother–Child Communication About CC

Two key facilitators will be discussed, namely conversations evolving as women discover the language to describe CC and as women develop strategies to discuss the father’s behavior with their children.

#### Subtheme 1: “These Are the Words That I Need”: Finding Language to Describe CC

Several mothers reflected on their journey to find the words to explain and discuss CC with their children. Language, therefore, seems to be experienced as a source of power, as a means of communication but also as a tool to reflect and shape social reality as women regain control over the narrative that had previously been controlled by abusive fathers.

*“It wasn’t until I read Evan Stark’s book about coercive control that I realised this, this is it. These are the words that I need. This man understands me. He's telling my life story. I get it now. . .I've got probably a lot stronger psychological understanding now as well I'm able to help them to make sense of it.”* (Dilys)

*“I've definitely, as I've discovered the language, and I think for me that's been a process. So as I've talked about things to him, there's been, sort of, my hesitancy, like is this really what I think it is? So I think that's been a progressive sort of explanation.”* (Nicola)

Women described gradually finding the language of “coercive control” through years of reading and research, having therapy, and support from domestic abuse organizations, and many women emphasized how they wish this information and language had been more easily accessible in order to facilitate these conversations much earlier.

#### Subtheme 2: “What Do You Think About That?”: Finding Ways to Talk About Dad’s Behavior

The women in this study had developed a number of strategies for talking with their children about the abuse, such as asking children open questions to encourage them to develop their own understanding of their father’s behavior. Given that mothers are often having these conversations in a context where children are constantly bombarded with a distorted version of reality by the abusive father, mothers reflected on how asking open questions allows children the space to think for themselves.

*“I'll say ‘okay, and what do you think about that?’. So I will kind of try to give her an opportunity to talk a little bit. . .And I think what I've seen is that then has given her a chance to kind of work something out and say, ‘well, I don't think that's right’.”* (Nicola)

Another strategy described was making conversations general, rather than specific to the father. Similar to the strategy of asking open questions, this may allow children to develop their own understanding of behaviors that are respectful, and those that are abusive.

*“I more recently have sort of tried to remove him as the person I'm talking about, but to make it more general, as in what makes a nice person? Or what would a kind person do in this situation?”* (Nicola)

*“I don't think I'm gonna make it like about her dad, ‘oh your dad did all of this’, but just tell her that, you know. . .no one should ever make you feel like you can't hang out with your friends, no one should ever, you know, bring you down, make you feel isolated.”* (Tamara)

### GET 5: Barriers of Mother–Child Communication About CC

The mothers in this study experienced far more barriers to talking openly with their children about CC than facilitators, and three key barriers are discussed: experiences of self-doubt in explaining the insidious and nuanced abuse tactics that make up CC, talking with children under the gaze of family court, and feeling unsupported in talking to children.

#### Subtheme 1: “Was It Even That Bad?”: Explaining the Insidious and Nuanced Abuse Tactics That Make Up CC

A key barrier to mother–child communication was mothers’ experience of self-doubt in relation to the complex, insidious, and often “unseen” abuse tactics that make up CC. Perpetrators of CC use tactics of denial, minimization, justification and gaslighting to deliberately distort reality (Katz, 2022). For example, Elizabeth describes being told by the father to *“get over yourself”* after she objected to him verbally abusing their children, saying *“it’s only language”*, with Mia describing how she was told to *“just stop making a big deal out of it.”* When trying to talk with children about the abuse and explain what happened, several mothers seemed to experience a sense of self-doubt, or perhaps internalized minimization, particularly in the absence of physical violence.

*“I didn't have the words to put to it at that time, you know, just thinking very much it was emotional, psychological, sexual, financial, massively financial abuse, um, but I always saw myself as being the lucky one because I hadn't experienced any physical abuse. So I think it was quite hard to put into words to the kids what had even, how to even explain it to them.”* (Dilys)

Dilys’ experience not only speaks to a sense of self-doubt and perhaps internalized minimisation, but also the complexity of talking with children about patterns of coercive and controlling behaviors, as opposed to isolated incidents of physical violence.

*“When I’m trying to explain to them, this is what he did, and I’m thinking in my head, well that doesn't sound like anything. So what am I going on about? This doesn’t seem like it's the big thing that it was. So yeah, death by a thousand pinpricks, it literally is. I think that’s why people find it so hard to explain coercive control cos it’s these little things that seem like nothing but, in actual fact, when you put them together it is a big picture.”* (Dilys)

These quotes powerfully depict the complexity in explaining CC to children; not only does the abuse leave women with a sense of uncertainty and self-doubt around what happened, but also explaining the hidden and nuanced dynamics of CC to children by focusing on specific incidents does not capture the cumulative effects of “*a thousand pinpricks*,” which further feeds the self-doubt and uncertainty during these conversations.

#### Subtheme 2: “The Worst Fear Is That They’ll Take My Son Away From Me”: Talking With Children Under the Gaze of Family Court

Almost all participants who had been involved in family court proceedings reflected on the fear they felt during conversations with their children related to how these conversations may be weaponized by the father within family court. For many women, they described the fear of being accused of PA as the biggest barrier to talking honestly with their children about the abuse, ultimately fearing that such an accusation may result in their children being removed to the sole custody of the abusive father.

*“I’m terrified that, you know, [ex-husband] is already saying that I’m ‘alienating’ them. . .his barrister yesterday said, ‘well they've only heard the mother's narrative for the last year’. She said that to the family court. So that is the biggest thing I feel which is hard.”* (Elizabeth)

*“Ultimately, the worst fear is that they'll take my son away from me. . .And we live in fear. I'm absolutely petrified at the moment. Absolutely petrified. . .Each conversation, for me, it makes me feel nervous. Am I allowed to tell him this? Am I saying the right thing? What are the consequences going to be?”* (Charlotte)

Several women reflected on the fear of the legal consequences of talking to children given how family court orders are written to prevent “denigration” of the other parent and are in fact written to instruct parents to be “*encouraging and supportive*” (Mia) of the other parent, leaving mothers with the impossible task of navigating how to talk honestly with children about the abusive father under these formidable court orders.

#### Subtheme 3: “A Lonely Little Island”: Feeling Unsupported in Talking to Children

Several women spoke about the wider lack of professional understanding about CC, which left them feeling alone in navigating these complex conversations.

*“It feels quite like a lonely little island where you're kind of on your own to deal with it. . .If the courts don't understand and the police don’t understand coercive control, which they don't by the way, just to be crystal clear. . .then there’s no support systems in place to facilitate those conversations or to encourage them.”* (Gemma)

*“[Answering whether anyone has supported her in talking to the children] No, I had to educate the social worker on what coercive control was.”* (Dilys)

Many women reported that no professional had ever supported them in talking with their children about the abuse. Nicola reflects on how she was once provided with a book to go through with her daughter about domestic violence, but that it very much focused on “*the noises that would happen in the house*,” which, therefore, did not apply to her daughter’s experiences of post-separation CC as Nicola had separated from the father before her daughter turned one. Not only feeling unsupported, but Mia comments on being actively discouraged from talking to her daughter about the abuse, describing how “*there’s such a huge pressure on parents to not involve children in ‘adult issues’.*”

## Discussion

This study aimed to explore mothers’ experiences of their relationships with their children in the context of CC, and mothers’ experiences of talking with their children about the CC. Through analyzing interview data from 11 participants using IPA, five group experiential statements were constructed, with 12 group-level subthemes.

There was a shared experience among the mothers in this study of closeness with their children despite their experiences of abuse, which mothers discussed in relation to trust and a sense of closeness through shared experiences. Given the proliferation of studies reporting negative associations between domestic abuse and mother–child attachment security (Chiesa et al., 2018; McIntosh et al., 2021), it is important to highlight mothers’ experiences of closeness with their children. Buchanan (2018) explores the many ways in which women used their agency to protect and find space to relate peacefully with their babies in situations of domestic abuse, arguing that attachment theory “exacerbates the underlying causes of domestic violence by accepting a deficit view of women” (p.126), with limited consideration of the context of sustained hostility in which women are parenting. It is a testament to the mothers in this study that they were able to maintain such trust and closeness with their children in the context of the continuous malignant presence of the father in the mother–child relationship. Similar to previous research, the women in this study experienced a plethora of abuse tactics post-separation, including intimidation, manipulation, stalking, and financial abuse (Katz, 2022; Tutty et al., 2023). This highlights the importance of the post-separation context and the need to continue to challenge the widely held assumption that the abuse ends at the point of separation.

In terms of mothers’ experiences of talking with their children about the abuse, overall mothers experienced these conversations as positive for the mother–child relationship. Mothers experienced these conversations with their children as facilitating sense-making, which is supported by previous research, which has highlighted how talking about the abuse helped children to understand what had been happening and help both mothers and children to break free from the distorted reality created by perpetrators/fathers (Katz, 2022; McGee, 2000; Moulding et al., 2015; Mullender et al., 2002). It is important to note the wide age range of women’s children (5–27). IPA aims for sample homogeneity in order to explore a specific experience in detail. While there was substantial convergence in women’s accounts, it may be that future research would benefit from focusing on narrower age ranges in order to explore the impact this has on language and communication around CC, such as whether mothers with very young children adopt more play-based approaches to talking about the abuse. Extending previous research, the current study also positions these conversations as facilitating trust between mothers and children. Katz (2022) emphasizes how the question of how mother–child relationships recover in the aftermath of CC is vital yet under researched. Talking honestly about the abuse, and providing the space to listen, seemed to be an important recovery process for several mothers in this study who experienced these conversations as the most helpful act in strengthening their relationships with their children post-separation.

The mothers in this study experienced several facilitators of these conversations, namely gradually learning the language needed to explain CC as well as the development of strategies that encouraged children to form their own understanding of their father’s behavior. Many participants reported barriers to mother–child communication about CC, noting that discussions on this topic were often absent or discouraged by professionals. Despite earlier calls to address the “conspiracy of silence” surrounding domestic abuse (McGee, 2000; Mullender et al., 2002), many felt unsupported in navigating these complex conversations. Women struggled to explain the insidious and nuanced abuse tactics that make up CC. Furthermore, as perpetrators/fathers have often minimized the abuse (Katz, 2022), creating a narrative where it “wasn’t really that bad,” especially in the absence of physical violence, this again hinders mother–child communication that facilitates a shared understanding of the abuse.

A significant barrier described by participants was the fear of how conversations with their children would be weaponised within family court. Being accused of PA was a threat that hung over mothers as they attempted to speak honestly with their children about the perpetrator’s/father’s behaviors. Such fears were not unfounded; Birchall and Choudhry (2022) explored the experiences of women survivors of domestic abuse in family courts in England and found that allegations of PA were frequently used during child arrangement proceedings to obscure and undermine allegations of domestic abuse. In fact, over a third of women in Birchall and Choudhry’s (2022) study had had their children removed to the perpetrator/father because of PA allegations. PA was effectively used to silence and disempower women, as echoed in recent research by Dalgarno et al. (2024). Barnett (2020) draws attention to the worrying growing acceptance of PA within English and Welsh family courts, alongside concerning increase in PA “experts” within court proceedings. Such “experts” have been quoted as invoking the work of Richard Gardner, the original author of “PA syndrome,” whose work has now been completely discredited within the scientific community (Barnett, 2020), yet continues to reverberate within U.K. family courts, unsurprisingly to the detriment of mothers.

### Implications for Clinical Practice and Future Research

#### Joint Mother–Child Interventions

Joint mother–child interventions promoting opportunities to talk about their shared experiences of abuse should be prioritized within the field of domestic abuse. Indeed, the National Institute for Health and Care Excellence (2014) recommends that interventions are provided to children who have experienced domestic abuse focused on supporting the relationship between the child and the protective parent. The National Society for the Prevention of Cruelty to Children has developed the Domestic Abuse Recovering Together program, which aims to strengthen mother–child relationships following domestic abuse and improve outcomes for children. Preliminary findings indicated that mothers and children particularly valued the joint sessions which provided opportunities to talk about the abuse (McManus et al., 2013; Smith et al., 2015). From an attachment perspective, Lieberman et al. (2015) advocate for joint mother–child interventions following domestic abuse, namely Child–Parent Psychotherapy, as the child’s developmental growth is primarily supported through their relationship with the protective parent, and so the mother–child relationship should be a key focus of intervention. Future research would benefit from a mixed-methods approach to investigate quantitatively the impact of mother–child communication about CC on mothers, children, and the mother–child relationship. Such research should capture children’s experiences of talking with their mothers about CC, as the current study is limited to mothers’ experiences only, as well as longer-term patterns of mother–child communication as children grow into adulthood.

#### Education and Training to Professionals

More training is needed to educate health and legal professionals on the “conspiracy of silence” that can surround domestic abuse and strategies to facilitate open communication between mothers and their children. Training is needed on the complex and insidious abuse tactics post-separation, which includes continued abuse and control via the weaponizing of conversations between mothers and children about the abuse. All professionals involved in family court proceedings need to understand how PA can be used by abusive men to attack mother–child relationships and silence women from talking with their children. Such training is vital in improving professionals’ confidence in working jointly with mothers and children to talk through experiences from the past, as lack of clinician confidence has previously been reported as a barrier to mother–child communication in the aftermath of domestic abuse (Humphreys et al., 2011).

#### Education in Schools

Education is needed to equip young people with the language needed to talk about CC. Since September 2020, relationships education has been compulsory for all primary schools, and relationships and sex education for all secondary schools (Department of Education, 2019). However, clearer guidance is needed that delineates key topics that should be covered, namely education on the array of abuse tactics used by perpetrators, including non-physical aspects of abuse, which would equip children with the awareness and language needed to identify and talk about patterns of CC. This is particularly important in the context of recent findings from the World Health Organization about the alarmingly high rates of intimate partner violence among adolescent girls (Sardinha et al., 2024).

#### Family Court Reform

The current study adds to calls for urgent reform of U.K. family courts (Birchall & Choudhry, 2022; Dalgarno et al., 2024; Katz, 2022), including “robust training on domestic abuse for all judges, magistrates and professionals in the family courts and access to legal aid for all parties within domestic abuse cases” (Women’s Aid, 2024). Several proposed legal changes have recently been debated in the House of Lords, including a ban on unregulated PA “experts.” This, and the use of PA, should be prohibited, as advocated by the United Nations (2023) and European Parliament (2022).

### Strengths and Limitations

In adopting a phenomenological approach, the current study contributes novel insights regarding mothers’ experiences of their relationships with their children and of talking with their children about the abuse, which is a useful addition to the current literature and helpful for practitioners working with women and children survivors. The involvement of experts by experience is a strength of the study, although due to time and financial constraints, the level of involvement was limited to consultation rather than collaboration or co-production. The current study is limited by the lack of inclusion of people of the global majority, as the sample was primarily comprised of White British women. Despite efforts made to recruit a more representative sample, this appeared insufficient to challenge long-standing structural barriers preventing people from a broad range of backgrounds from research participation. Future research may benefit from a sampling strategy that intentionally includes people from a broader range of backgrounds with different social characteristics. It is also important to acknowledge the focus on heterosexual relationships. Future research should aim to explore the perspectives of people from specific groups within the LGBTQ community, whose experiences are likely to differ to those of women abused by male partners. A final limitation is that the current study was not able to capture children’s experiences of their relationships with their mothers and talking with their mothers about the abuse.

## Conclusion

The current study provides valuable insights into how mothers experience their relationships with their children in the context of CC and engage in conversations about the abuse. It highlights the complex ways in which mothers experience both closeness and strain in their relationships with their children, as coercively controlling fathers actively seek to undermine these connections. Mothers showed considerable thoughtfulness and sensitivity around how to discuss the abuse with their children, often finding these conversations to be beneficial for the mother–child relationship. The findings contribute to a growing evidence base of the importance of supporting conversations between mothers and their children in relation to their experiences of abuse in order to strengthen mother–child relationships post-separation. Future efforts should prioritize supporting these conversations as a crucial step toward recovery for families affected by CC.
